# Nucleic Acid-Based Detection of *Pythium insidiosum*: A Systematic Review

**DOI:** 10.3390/jof9010027

**Published:** 2022-12-23

**Authors:** Thanawat Sridapan, Theerapong Krajaejun

**Affiliations:** Department of Pathology, Faculty of Medicine, Ramathibodi Hospital, Mahidol University, 270 Rama VI Road, Bangkok 10400, Thailand

**Keywords:** pythiosis, *Pythium insidiosum*, oomycete, diagnosis, nucleic acid, molecular detection

## Abstract

Pythiosis, a life-threatening infectious condition caused by *Pythium insidiosum*, has been increasingly reported in humans and animals worldwide. Antifungal drugs usually fail to control the pathogen. The surgical removal of an infected organ is the treatment of choice. Many affected patients die due to advanced infection. A timely and accurate diagnosis could lead to a better prognosis in pythiosis patients and save their lives. Although a standard culture method is available in microbiological laboratories, it is time-consuming, laborious, and insensitive for *P. insidiosum* identification. Immunological assays have been developed to improve the diagnosis of pythiosis. However, immunological methods are commercially unavailable and primarily detect anti-*P. insidiosum* antibodies, which constitute indirect evidence of pythiosis, making it challenging to differentiate a past from a recent infection. Moreover, such immunological tests cannot diagnose patients with a local infection, such as in the eye. Nucleic acid-based tests (NATs) are efficient for the direct and rapid detection of *P. insidiosum* DNA in trace-amount or culture-negative specimens. The reagents and equipment required for NATs are usually available in molecular diagnostic laboratories. Herein, we provide a systematic review to comprehensively present the principal and clinical usages, advantages, and limitations of such NATs in the detection of *P. insidiosum*. Various NATs have been established to detect *P. insidiosum*, which can be classified into amplification-based (i.e., PCR assays, isothermal tests, and next-generation sequencing methods) and non-amplification-based (i.e., DNA hybridization) techniques. This concise review on NATs constitutes an up-to-date reference with which healthcare professionals can learn about and decide upon which detection method is suitable for their respective laboratory environments.

## 1. Introduction

Pythiosis is a life-threatening infectious disease of humans and animals caused by the pathogenic oomycete *Pythium insidiosum* [1]. The infection has been primarily reported in tropical, subtropical, and temperate countries [1,2]. Cutaneous/subcutaneous lesions and gastrointestinal disorders are typical clinical features reported in affected animals. In contrast, arterial occlusions of the lower extremities and ocular infections are predominant presentations in affected humans, particularly those with hematological disorders, such as thalassemia hemoglobinopathy [1,2]. Medical therapy using conventional antifungal drugs usually fails to control a *P. insidiosum* infection. The surgical removal of the infected organ (i.e., the eye or leg) is the treatment of choice for pythiosis. Many pythiosis patients die due to advanced infection or inadequate treatment [3]. A delayed diagnosis leads to late treatment and a poor prognosis in pythiosis patients [3,4]. 

The conventional method for diagnosing pythiosis is a microbiological culture to isolate *P. insidiosum* from infected tissue. Such a laborious and time-consuming method requires experienced personnel to perform the procedure and interpret the result [1,5]. Moreover, it often fails to isolate the organism, which may be sparsely present in a tissue sample and could die at low temperatures during the sample’s transportation and storage [1,6,7]. Therefore, a more sensitive method is needed to facilitate the early detection of *P. insidiosum* and the prompt treatment of affected patients. Several alternative detection methods, relying on sensitive and specific immunological and molecular technologies, have been implemented to overcome the microbiological culture method’s limitations in identifying *P. insidiosum* [5,8]. Although the immunological methods developed so far are rapid and efficient procedures, it is often difficult to indicate whether the detected antibodies in the patient’s blood are from a past or recent *P. insidiosum* infection. Moreover, an immunological test cannot detect anti-*P insidiosum* antibodies in patients with a local infection, such as in the eye, which is an immunological privilege site that lacks certain types of host immune response (i.e., antibody production) [9,10]. No immunological test for pythiosis is commercially available. Moreover, the in-house development of such a test requires a *P. insidiosum*-specific antigen. The unavailability and inaccessibility of an immunological test prevent its usage in clinical laboratories. 

Considering another diagnostic modality, the detection of the DNA or RNA sequence specific to a target pathogen by nucleic acid-based tests (NATs) is an effective procedure for reliable microbial identification [8,11]. Once a pathogen’s nucleic acid sequence is known, probes and primers can be designed and synthesized for specifically hybridizing it. Molecular detection techniques using such probes or primers can rapidly capture a trace element of the pathogen’s nucleic acid in a clinical specimen, highlighting its diagnostic sensitivity. Unlike the in-house immunological assay, all critical reagents (i.e., primers, probes, nucleotides, and DNA polymerases) and necessary equipment required for developing NATs are readily available in molecular diagnostic laboratories, or they can be acquired from a commercial source. 

During the past decades, various NATs have been established for detecting *P. insidiosum*. Since published details on these *P. insidiosum*-detecting NATs are scattered, we performed a systematic review to collect and summarize the current information on the principles and application of such assays, including the recently developed next-generation sequencing (NGS) technology. Moreover, the advantages and limitations of each assay are provided, making it convenient for healthcare professionals to learn and decide which detection method is suitable for use in their respective laboratory environments. It should be noted that the specific genotypes (i.e., clade III) of *P. insidiosum* have recently been proposed as a new species called *P. periculosum* [12]. However, to avoid confusion, we only referred to the organism as *P. insidiosum* in this study, as do all the collected publications regarding NATs.

## 2. Methods

### 2.1. Study Search and Selection

We conducted a systematic review to collect the studies on *P. insidiosum*-detecting NATs deposited in 2 public databases, PubMed (https://pubmed.ncbi.nlm.nih.gov; accessed on 30 September 2022) and Google Scholar (https://scholar.google.com/; accessed on 30 September 2022 ), following the Preferred Reporting Items for Systematic Reviews and Meta-Analyses (PRISMA) guidelines [13]. The keywords for searching the relevant studies were “*Pythium insidiosum* OR *Pythium* sp. OR pythiosis” AND “molecular OR analysis OR diagnosis OR detection OR identification OR differentiation OR evaluation OR sequencing OR genome sequence”. Regarding inclusion criteria, all studies that described the detection and identification of *P. insidiosum* by NATs were gathered in the collection. Regarding exclusion criteria, irrelevant original articles that reported non-nucleic-acid-based diagnostic approaches (such as microbiological culture, histological assay, immunodiagnostic test, and proteomic analysis) and studies without available full text were removed from this review. The reference list of each recruited article was manually checked to find any other studies describing a *P. insidiosum*-detecting NAT not identified from PubMed and Google Scholar searches.

### 2.2. Data Extraction

Duplicate publications from the literature search were removed. One researcher (T.S.) screened the titles and abstracts to collect only the reports on NAT for *P. insidiosum*. The other researcher (T.K.) double-checked the relevance of the selected studies. The full texts of the recruited articles were read in detail and further evaluated against the inclusion and exclusion criteria to obtain the final set of publications used in this study. Information about the principles and applications of the NATs for *P. insidiosum* was extracted and summarized for the presentation and visualization of the data. The test principles of NATs for *P. insidiosum* were divided into non-amplification and amplification techniques, as defined by Mothershed and Whitney [14]. Moreover, the advantages and limitations of each NAT were gathered and discussed in this review. 

## 3. Results and Discussion

### 3.1. Study Recruitment

**Figure 1:**
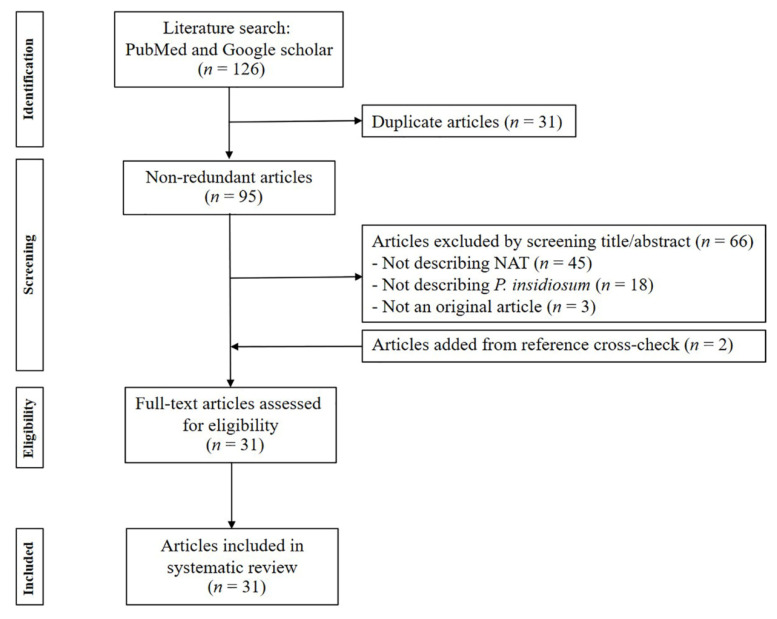
PRISMA-based flow chart demonstrating the article-procurement process.

After removing 31 duplicate articles, 95 non-redundant articles were identified from 2 databases (i.e., PubMed and Google Scholar) using the defined keywords (see the Section 2). Reviewing the titles and abstracts showed that 29 articles were relevant to NATs for *P. insidiosum*. Manual checks of the reference lists in the collected articles identified two additional articles reporting *P. insidiosum*-detecting NATs. Adhering to the inclusion and exclusion criteria (see the Section 2), a total of 31 articles published during 2001–2022 reported the detection and identification of *P. insidiosum* using NATs. The final publication list was subjected to data extraction to describe the principles, applications, advantages, and limitations of NATs for detecting *P. insidiosum*. A PRISMA-based flow chart demonstrating the article-procurement process is shown in Figure 1.

### 3.2. Classification, Principles, and Applications of P. insidiosum-Detecting NATs

The general principle of NATs relies on detecting organism-specific DNA or RNA sequences. This diagnostic modality is suitable and efficient for clinical microbial detection due to its high specificity, sensitivity, and short turnaround time [14,15]. It can also be used to detect a pathogen of interest in a clinical specimen, even though such a sample is sometimes culture-negative [14]. Several different types of NATs have been developed for microbial detection, which can be classified into (i) nucleic acid hybridization (non-amplification) and (ii) nucleic acid amplification tests (NAATs) [14]. The non-amplification technique does not multiply the target sequence because the target organism needs to be culture-isolated from or present in a significant amount of the specimen before performing the detection procedure. Such technology includes Southern blot, Northern blot, and dot-blot hybridization. In contrast, NAATs amplify a minimal amount of the genetic material of an organism until up to one billion copies are obtained, with no prior microbial isolation (culture) required. These amplification techniques allow for the detection of an organism that is difficult to grow via traditional microbiological methods or when the pathogen itself is present in trace amounts. NAATs can be further categorized into three major types: PCR-based, isothermal amplification-based, and NGS-based approaches.

**Figure 2:**
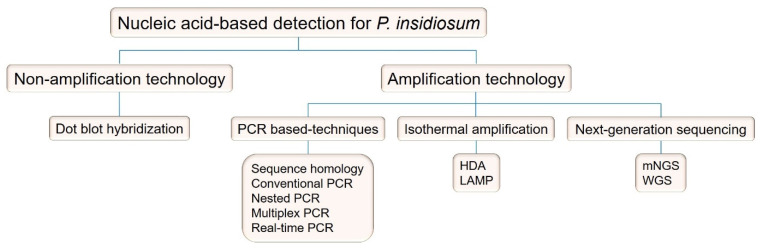
Nucleic acid-based tests established for *P. insidiosum* detection. Abbreviations: PCR, polymerase chain reaction; HDA, helicase-dependent amplification; LAMP, loop-mediated isothermal amplification; mNGS, metagenomic next-generation sequencing; WGS, whole-genome sequencing.

Many NAT techniques have been established to facilitate the detection of *P. insidiosum*. These techniques can be classified into two groups, namely, (i) non-amplification and (ii) nucleic acid amplification, as shown in Figure 2. The principles and applications of the *P. insidiosum*-detecting NATs are summarized below. Moreover, the primer sequences referred to in each NAT are listed in Table 1.

#### 3.2.1. Non-Amplification Technology: Nucleic Acid Hybridization

Nucleic acid hybridization can identify specific DNA or RNA without target sequence amplification [49]. This method relies on forming nucleic acid duplexes between two complementary strands of a designed oligonucleotide probe and the target nucleic acid sequence [50]. A positive hybridization signal is obtained when the probe sequence perfectly anneals the target sequence. A probe-to-target-hybridized formation can be detected by a radioisotope or fluorescence molecule tagged in the probe [51]. Various hybridization formats, such as Northern blot, Southern blot, and dot/slot blot, have been used in modern laboratories [52]. Among these nucleic acid blots, the dot-blot hybridization technique has been developed for detecting *P. insidiosum* [53]. 

Dot-blot hybridization is a nucleic acid-based detection technique for high throughput or large-scale sample testing. It relies on the direct and semi-quantitative detection of a specific nucleotide sequence dotted on a membrane without prior electrophoresis-based separation and gel-to-membrane transference of the target sequence [54]. Technically, the target DNA or RNA sample extracted from the organism or cell in a specimen is spotted onto a membrane before hybridization by a labeled nucleic acid probe. On the other hand, the probe can be pre-blotted onto a filter membrane before hybridizing it with a corresponding target sequence, giving rise to a technique called “reverse dot blot”. 

**Figure 3:**
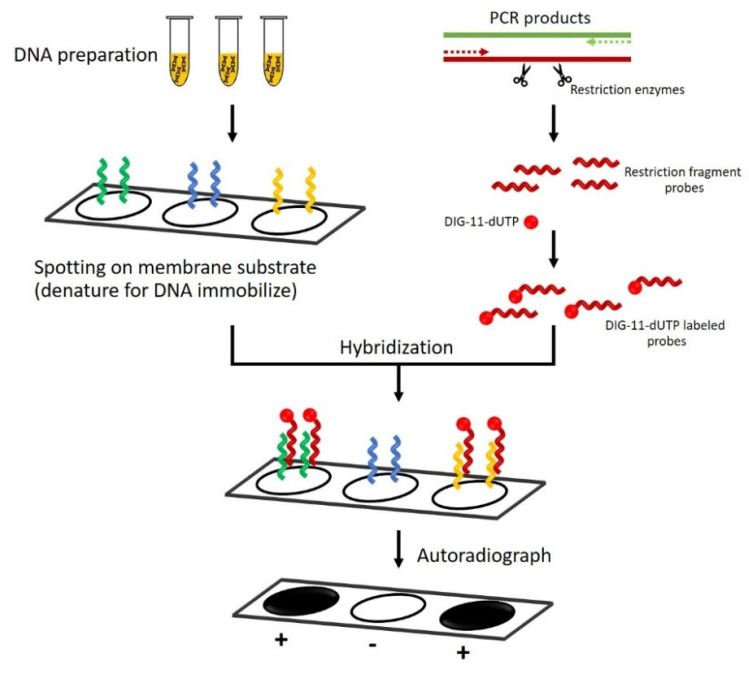
Diagram of the dot-blot hybridization technique for detecting a target nucleic acid sequence of *P. insidiosum*. Different DNA samples (represented by green, blue, and yellow curly lines) are extracted from the organism and blotted onto a membrane (the left arm of the diagram). A DNA probe (represented by a red curly line) is prepared by using a restriction enzyme and labeled with a chemiluminescent molecule (i.e., digoxigenin (DIG)-11-dUTP; represented by a red dot) to generate a detection signal (the right arm of the diagram). The blotted target DNAs are denatured into single strands by heat or alkali treatment before hybridizing them with the DIG-11-dUTP-labelled probe. After removing unbound radioactive probes through a washing step, the hybridized membrane is subjected to X-ray film exposure. Only a sample containing a DNA complementary sequence with the labeled probe shows a black spot on the film, indicating a positive result.

Schurko et al. were the first to develop the dot-blot hybridization technique for the detection of *P. insidiosum* [53], as depicted in Figure 3. They constructed species-specific probes using a 530 bp fragment derived from *Hinf*I site-specific deoxyribonuclease digestion of the intergenic spacer I (IGS-1), located between the LSU and 5S rRNA sequences of *P. insidiosum*. The hybridized target sequence of the pathogen can be detected by a digoxigenin (DIG)-11-dUTP-labeled probe that generates a chemiluminescent signal on X-ray film. By evaluating 104 different *Pythium* species and some other pathogenic microorganisms, the detection probe specifically hybridized target DNA samples extracted from all 22 genetically diverse isolates of *P. insidiosum* without cross-hybridization with the other organisms tested. However, Schurko et al. only tested the performance of this detection technique against the *P. insidiosum* DNA extracted from pure cultures [53]. Future evaluation of this established technique should be focused on infected tissues in order to prove the test’s efficiency for detecting *P. insidiosum* in the clinical setting. Nevertheless, apart from its multistep procedure and relatively high cost, dot-blot hybridization using the *P. insidiosum*-specific probe requires trained personnel to perform the assay, which could limit its routine uses in clinical laboratories.

#### 3.2.2. Nucleic Acid Amplification Technology

##### PCR-Based Techniques

**Figure 4:**
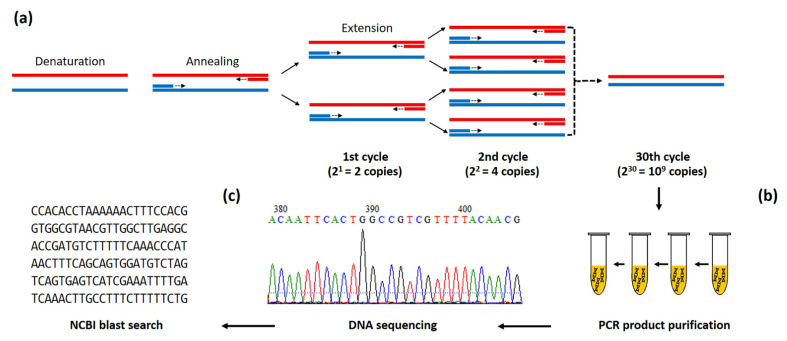
Scheme of the PCR amplification and sequence homology analysis for detecting *P. insidiosum*. (**a**) Conventional PCR amplification relies on target-specific forward and reverse primers and a repeated 3-step process, which includes denaturation, annealing, and extension. This PCR process is repeated for 25–30 thermal cycles, resulting in exponential amplification of a single target sequence to up to millions of copies. (**b**) After a purification step, the PCR product is subjected to direct DNA sequencing. (**c**) The obtained sequence is then BLAST-searched against the up-to-date NCBI database (https://blast.ncbi.nlm.nih.gov/Blast.cgi, accessed on 6 November 2022) to identify a significantly matched homology sequence—the final step for microbial identification.

PCR was first invented in 1985 by Mullis and colleagues [55]. This DNA amplification technique has become a popular fundamental procedure used in various molecular applications, such as pathogen detection, inherited disease diagnosis, and sample preparation for downstream experiments (i.e., DNA sequencing and gene cloning) [8]. The PCR technique relies on a set of designed primers used to amplify a specific target sequence in a repeated three-step process, as depicted in Figure 4a. In the first step (denaturation), a target double-stranded DNA is denatured into single-strands by heat (94–98 °C). Then, in the second step (annealing), which takes place at 55–70 °C, a primer pair specifically anneals the denatured target DNA. The last step (extension) involves the polymerization or synthesis of a new DNA strand in the presence of deoxyribonucleotides and a thermostable DNA polymerase at the desired temperature (i.e., 68–72 °C). This three-step process is repeated for ~30 cycles by a thermocycler machine, leading to the exponential amplification of a few target sequences into millions of copies [11]. After staining with an intercalating DNA dye (i.e., ethidium bromide), a PCR product (amplicon) can be visualized as a size-expected band on an electrophoresis gel [14]. The amplicon can be further processed for DNA sequencing and homology analysis as part of a standard microorganism identification procedure. Various PCR-based techniques have been successfully developed for detecting *P. insidiosum* DNA in a clinical specimen or pure culture. Such techniques include sequence homology analysis [37], conventional PCR [36,47], nested PCR [16,17,32,40,41], multiplex PCR (m-PCR) [19,35], and real-time/quantitative PCR (qPCR) [46,48], as described in detail below.

(1) Sequence homology analysis

The PCR amplification of a target gene followed by the direct sequencing and homological search of an obtained DNA sequence against a public database (Figure 4) is a widely used standard procedure (termed sequence homology analysis) for the identification of various microorganisms [56], including *P. insidiosum* [37]. Such an analysis aims at identifying a significantly matched homologous sequence of an organism in question against the up-to-date GenBank database using the Basic Local Alignment Search Tool (BLAST) available online at the National Center for Biotechnology Information (NCBI) website (https://blast.ncbi.nlm.nih.gov/Blast.cgi, accessed on 6 November 2022) [56]. A commonly used target sequence is ribosomal DNA (rDNA; alternative name: ribosomal RNA (rRNA) gene repeat), which comprises 18S ribosomal RNA, an internal transcribed spacer (ITS) 1, 5.8S ribosomal RNA, ITS2, and 28S ribosomal RNA [17]. 

Sequence homology analysis is an effective strategy that can be performed in a general molecular laboratory in order to identify and confirm the presence of *P. insidiosum* in a sample isolated from a pythiosis patient. Badenoch et al. used the universal fungal primers NS1 and NS2 [23] to amplify a 510 bp 18S rRNA portion from the genomic DNA of an organism isolated from a patient with an unknown ocular infection [37]. A BLAST search showed that the obtained amplicon sequence matched an oomycete organism of an undefined species. Further, PCR and sequence analyses using the primers TW81 and AB28 successfully amplified a 900 bp amplicon from the internal transcribed spacer, including ITS1, the 5.8S gene, and ITS2 regions [38]. The obtained sequence showed 99.0% homology with the *P. insidiosum* reference strain CBS777.84, thereby successfully identifying an unknown etiologic pathogen. 

Afterward, many investigators employed this sequence homology analysis strategy, using different sets of primers, to reach definitive diagnoses of pythiosis. For example, Reis et al. [42] confirmed a *P. insidiosum* infection in a horse by sequencing the amplified 18S rRNA sequence using the universal fungal primers NS1 and NS8 [23]. Vanittanakom et al. sequenced a 580 bp 18S rRNA-derived PCR product using the primers Pin1 and Pin2 and, consequently, identified *P. insidiosum* isolated from a human case [36]. Rivierre et al. reported the first subcutaneous pythiosis case in a dog from Africa [18], which was confirmed by the sequence homology analysis of a 785 bp ITS region amplified by the universal fungal primers ITS1 and ITS4 [23]. Mendoza et al. employed this technique using the primers NS1 and NS8 to diagnose the first case of canine pythiosis in Venezuela [43]. Bosco et al. [34] detected a *P. insidiosum* infection in a Brazilian horse by amplifying and analyzing a 900 bp rDNA sequence using the primers ITS4 and ITS5 [23]. Salipante et al. [20] used the same technique to diagnose pythiosis in a pediatric patient [20]. Using the primers ITS1 and ITS2 [30], they amplified and sequenced the ITS1 region of the genomic DNA extracted from formalin-fixed, paraffin-embedded (FFPE) tissue. The obtained sequence matched the reference *P. insidiosum* sequences deposited in GenBank. The pathogen sequence was amplified and further confirmed using additional FFPE tissue blocks and primers targeting ITS1 and *cox*1 sequences [44]. Using the method described by Tarai et al. [31], Sharma et al. sequenced the ITS1-5.8S-ITS2 regions of corneal tissue-extracted DNA samples from 162 patients with unknown keratitis; consequently, *P. insidiosum* DNA could be detected in 13 samples [21]. Konradt et al. [28] confirmed the diagnosis of cutaneous pythiosis in cattle in southern Brazil using the primers ITS1 and ITS4 [23]. 

Besides its clinical diagnostic applications, sequence homology analysis has been used to survey the presence of *P. insidiosum* in the environment. For example, Supabandhu et al. [29] confirmed the presence of *P. insidiosum* in 59 organisms isolated from rice paddy fields, irrigation channels, and water reservoirs by sequencing the ITS regions using the primers ITS1/ITS4 [23] and ITSpy1/ITSpy2 [36]. As mentioned above, sequence homology analysis has been well-accepted as an effective and reliable strategy for identifying *P. insidiosum.* However, as a downside, this technique involves multiple steps and is time-consuming, especially with respect to the sequencing process, which may take weeks to obtain a sequence for homology analysis, resulting in a delayed diagnosis.

(2) Conventional PCR

The conventional diagnostic PCR assay requires organism-specific primers, template DNA, PCR reagents, thermal cycler, and gel electrophoresis equipment to amplify and detect *P. insidiosum* DNA and reach a definitive diagnosis of pythiosis. A famous target sequence is rDNA [17]. Within several hours, the conventional PCR, using a pair of *P. insidiosum*-specific primers, can detect the pathogen in a biological sample without requiring DNA sequencing of the PCR product. The assay interpretation relies on the presence of a PCR product at the expected size on an electrophoresis gel. Vanittanakom et al. designed two specific primers (ITSpy1 and ITSpy2) that can detect *P. insidiosum* by conventional PCR [36]. Later, Keeratijarut et al. demonstrated that the *P. insidiosum* rDNA-specific primers ITSpy1 and ITSpy2 failed to amplify some isolates of *P. insidiosum* [47]. They introduced a new pair of primers (namely, Dx3 and Dx4) that targeted an alternative target gene: the exo-1,3-β-glucanase gene (*exo*1). Based on the DNA samples extracted from *P. insidiosum* isolates and various fungal species, the conventional PCR technique using the primers Dx3 and Dx4 showed 100% detection sensitivity and specificity [47].

(3) Nested PCR

**Figure 5:**
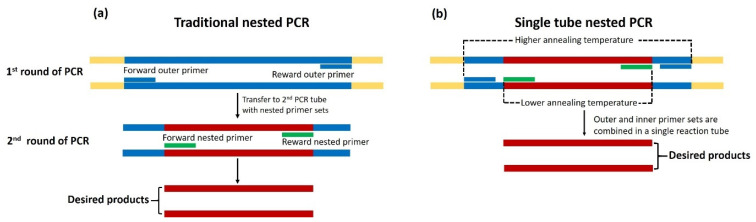
Schematic diagram of traditional and single-tube, nested PCR assays for detecting *P. insidiosum*. (**a**) The traditional nested PCR method requires two rounds of PCR amplification. The first-round reaction amplifies a target sequence using the first or outer primer pair (short blue lines). The amplicon from the first-round PCR serves as a template of the second or outer primer pair (short green lines) for amplifying the *P. insidiosum*-specific PCR product. (**b**) For the single-tube, nested PCR, the assay combines outer (short blue lines) and inner (short green lines) primers to amplify the target sequence in one tube. The first-round PCR, using the outer primers, is performed at a higher annealing temperature. In contrast, the second-round PCR, using the nested primers, is sequentially conducted at a lower annealing temperature. The diagram is adapted from the references [59,60].

A nested PCR assay is a modified version of the conventional PCR method. Technically, this amplification assay is designed to increase the detection sensitivity and specificity of a normal PCR reaction [57]. It consists of two sequential amplification reactions using two different primer pairs, outer and inner (nested) primers [14], as described in Figure 5a. The first-round amplification employs two outer primers to amplify a larger target fragment using a traditional PCR reaction. The product from the first-round PCR serves as a template for the second-round amplification using two inner primers designed to anneal the target sequence precisely [57]. This two-step strategy can enrich the target sequence (in the first-round PCR) and reduce non-specific amplification (in the second-round PCR), resulting in increased detection sensitivity and specificity [57]. Nested PCR has been widely used to detect organisms present in a low quantity. Grooters et al. successfully developed a nested PCR method for detecting *P. insidiosum* [17]. In the first-round amplification, they used the universal fungal primers ITS1 and ITS2 to amplify the rRNA sequence. Their second-round PCR employed the rRNA ITS1-targeting primers PI-1 and PI-2 in order to obtain a 105 bp amplicon from only DNA samples extracted from *P. insidiosum* but not from other control organisms, indicating the high detection sensitivity and specificity of the assay. However, Vanittanakom et al. showed that the inner primers PI-1 and PI-2 failed to identify one Thai *P. insidiosum* strain [36], proceeding to caution against the valorization of the detection sensitivity of such primers. Nevertheless, the nested PCR primers reported by Grooters et al. [17] can identify *P. insidiosum* in various clinical specimens, such as frozen and ethanol-fixed infected animal tissue [24]. Moreover, Botton et al. [16] demonstrated that the nested PCR is an efficient assay for detecting *P. insidiosum* in the tissue samples from horses (naturally affected hosts) and rabbits (experimental hosts) with pythiosis. Alternative primer sets, such as ITSpy1 and ITSpy2 [36], ITS1 and ITS2P [24], and ITS1 and ITS4 [16], could replace the use of the original outer primers ITS1 and ITS2 in the first-round nested PCR. 

As part of a survey of the *P. insidiosum* habitats in the environment, the nested PCR assay, using the outer primers ITS1 and ITS4 and the inner primers PI-1 and ITS2R, was able to identify the organism in question in irrigation water and soil samples in northern Thailand [22]. More recently, Elshafie et al. [32] developed another nested PCR method with which to detect *P. insidiosum* in FFPE-biopsied tissues from dogs. They amplified a 623 bp amplicon of the ITS2 non-coding region using the pan-fungal primers ITS3 and ITS4 [33]. The newly designed inner primers were then employed to amplify a 191 bp *P. insidiosum*-specific amplicon [32]. As per the report of Elshafie et al., the nested PCR technique could facilitate the diagnosis of pythiosis in dogs [32]. Although nested PCR is a highly specific and sensitive detection method, it is prone to carry-over contamination during the assay procedure [58]. For example, transferring the PCR product from the first- to second-round PCR reaction potentially leads to a false positive result. To address this issue, a single-tube, nested PCR assay has been developed by Thongsri et al. [7], in which two rounds of PCR amplification occur in the same tube, as depicted in Figure 5b. Two sets of nested PCRs were designed to target the 18S rRNA of *P. insidiosum*, with the annealing temperatures of 68 °C for the outer primers (i.e., CPL6 and CPR8) and 58 °C for the inner primers (i.e., YTL1 and YTR1). Moreover, the single-tube, nested PCR method has been used to detect *P. insidiosum* infection in patients with suspected fungal keratitis [40,41]. The single-tube, nested PCR method offers excellent sensitivity and specificity for rapidly detecting *P. insidiosum*—even in a trace amount of specimen.

(4) Multiplex PCR (m-PCR)

m-PCR is a variant PCR technique that utilizes more than one primer pair to simultaneously amplify multiple target sequences in a single reaction [8,11]. This technique reduces an assay’s duration, effort, and the use of costly polymerase and DNA templates compared with conventional PCR [61]. The m-PCR produces multiple amplicons with expected sizes that can be visualized by agarose gel electrophoresis. Designing the m-PCR primers is complicated because all the primers should have a similar annealing temperature, and the interaction between primers (i.e., primer dimers) should be minimal or absent [11]. The optimal concentration of each primer needs to be adjusted, ensuring a reliable amplification reaction and, thus, amplicon yield [11].

**Figure 6:**
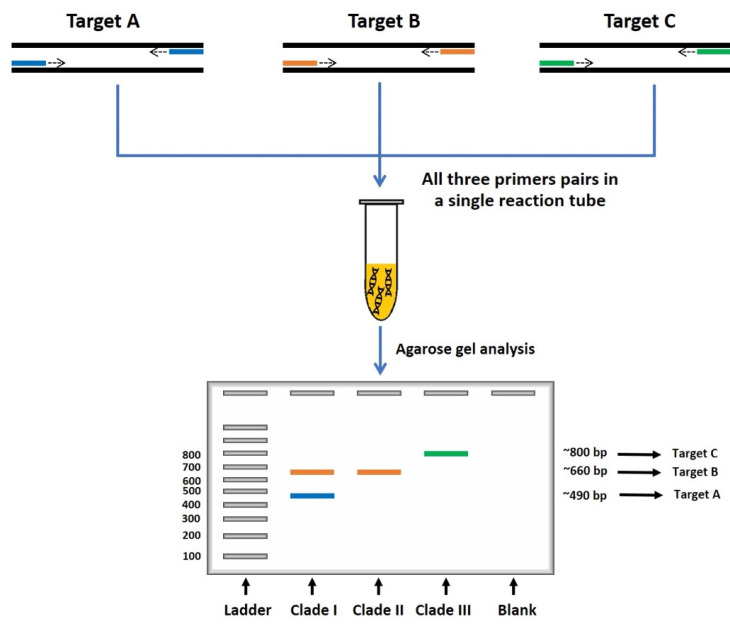
Schematic diagram of multiplex PCR assay for detecting and genotyping *P. insidiosum*. The multiplex PCR requires several pairs of primers (represented as short blue, orange, and green lines) to simultaneously amplify multiple target sequences (i.e., different rDNA sequence regions of *P. insidiosum*) in one reaction tube. The number and sizes of the resulting amplicon can be assessed by gel electrophoresis, which aided in identifying and genotyping the organism in question. The diagram is adapted from the references [19,59].

Rujirawat et al. [19] developed an m-PCR assay targeting the rDNA sequence in order to identify genetically diverse *P. insidiosum* strains. These investigators employed several genotype-associated single nucleotide polymorphisms (SNPs) presented within the rDNA sequence to design a common forward (namely, ITS1) and three reverse (namely, R1, R2, and R3) primers capable of detecting and, at the same time, classifying the organism into three distinct phylogenetic genotypes. The primers ITS1, R1, R2, and R3 together generate several amplicons with defined sizes (i.e., 490, 660, and 800 bp), in which the amplicon number and sizes define the *P. insidiosum* genotype, such as clade I (two amplicons of 490 and 660 bp), clade II (an amplicon of 660 bp), and clade III (an amplicon of 800 bp), as described in Figure 6. The m-PCR assay exhibited 100% detection sensitivity and specificity based on the performance assessment against 53 *P. insidiosum* isolates and 22 control fungi. The SNP-based m-PCR assay has been used to identify and genotype *P. insidiosum* isolated from affected animals (primarily horses and dogs) in South America [26] and Egypt [25], as well as in the environment in Thailand [27]. Recently, Kulandai et al. [35] described a duplex-PCR (d-PCR) assay for simultaneously detecting the 18S rRNA (using the primers Pin1 and Pin2) and ITS (using the primers ITSpy1 and ITSpy2 [36]) sequences of *P. insidiosum* in corneal specimens from patients with suspected ocular pythiosis. The d-PCR assay generated 580 bp and 233 bp amplicons from the *P. insidiosum*’s target sequences. Based on the samples analyzed by the investigators, the d-PCR assay showed 100.0% detection specificity and 92.8–100.0% detection sensitivity.

(5) Real-time or quantitative PCR (qPCR)

qPCR is an amplification technique for explicitly identifying and quantifying a nucleic acid material of interest in a biological sample [8]. qPCR offers several advantages, such as its ability to quantify amplified products; analyze the amplicon in real-time during DNA amplification; and detect multiple samples simultaneously. The reaction occurs in a closed system, within a single tube, and without exposing the amplified products to the laboratory environment, thereby minimizing carry-over contamination [15]. This method does not require agarose gel electrophoresis for an end-point analysis of the PCR product, leading to reduced assay duration and increased sample throughput [15]. The number of amplicons is measured in real-time at each thermal cycle using a non-specific intercalating fluorescent dye or a specific fluorescent dye-labeled probe [11]. The fluorescent signal appears proportional to the generated amplicon quantity. It should be noted that a non-specific fluorescent dye, such as SYBR Green, could intercalate in any double-stranded DNA materials, including primer dimers, contaminated DNA, and non-specific PCR products, possibly resulting in the readout of a false-positive result.

**Figure 7:**
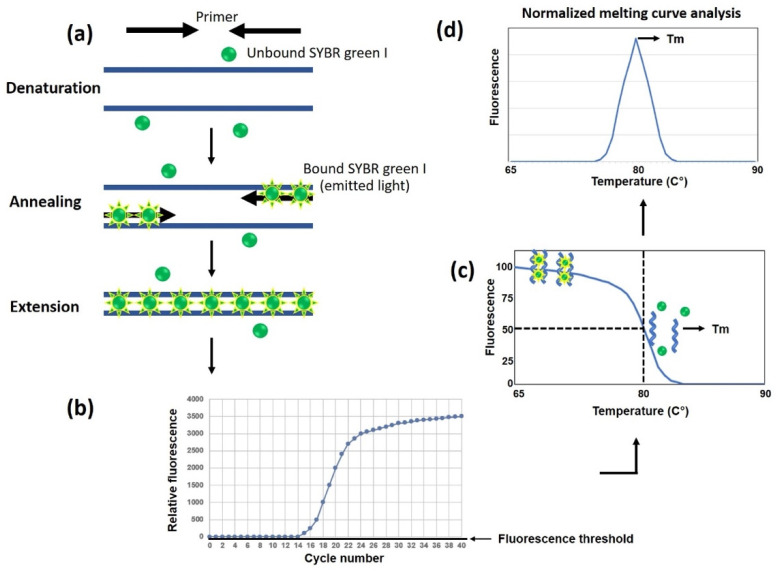
Schematic diagram of a real-time or quantitative PCR (qPCR) and melting curve analysis. (**a**) SYBR green, a non-specific fluorescent dye, binds all double-stranded, but not single-stranded, DNAs and emits a detectable fluorescent signal. Double-stranded DNA products accumulate during repeated thermal cycles, increasing SYBR green fluorescence emission. (**b**) The fluorescent signal in the reaction is proportional to the number of accumulated amplicons, which can be real-time-detected and displayed as an amplification plot. (**c**) When the PCR amplification is complete, a post-PCR dissociation/melting curve of the amplicons is analyzed to assess the assay’s specificity. At the initial phase of the curve, SYBR Green-incorporated double-stranded DNAs appear in a high proportion. An increased temperature denatures or melts the amplicons into single-stranded DNAs, resulting in SYBR Green’s dissociation and a decline in fluorescent signal. (**d**) Based on the obtained data, a normalized melting curve can be generated to show the melting point (T_m_) specific to the desired amplicon, wherein 50% of the double-stranded DNAs (primarily amplicons) are denatured. The diagram is adapted from the online reference real-time PCR handbook available online at http://www.gene-quantification.de, accessed on 6 November 2022.

Due to its extremely high detection sensitivity and specificity and relatively short turnaround time, qPCR has been widely used to detect many pathogens, including *P. insidiosum*, and has aided in the early diagnosis of infectious diseases. Keeratijarut et al. [48] developed a qPCR assay using SYBR Green as a non-specific intercalating fluorescent dye to rapidly detect *P. insidiosum* by targeting the *exo*1 gene, as depicted in Figure 7. The diagnostic performance of qPCR appeared to possess 100% sensitivity and specificity based on a sample set comprising 35 *P. insidiosum* isolates and 58 various control fungi. The analytic sensitivity (limit of detection) of qPCR is 1 × 10^−4^ ng gDNA template, as opposed to 1 ng for the conventional PCR.

The post-PCR dissociation (melting) curve is another qPCR characteristic that can be used to confirm whether the fluorescence signal is generated from the target sequence and not from non-specific PCR products. Worasilchai et al. [46] incorporated a type of post-amplification melting curve analysis, called “high-resolution melt analysis” (HRM), into a qPCR designed to target the *P. insidiosum cox*2 gene, giving rise to an assay, namely, qPCR-HRM. HRM can discriminate sequence variations based on sequence length, nucleic acid composition, GC content, and strand complementarity. Their assay rapidly detected and genotyped *P. insidiosum* and assessed the presence of non-specific amplicons and primer dimers. Based on the SNPs presented within the resulting 91 bp amplicons, qPCR-HRM can differentiate 15 *P. insidiosum* isolates from 28 of the related organisms tested. Moreover, HRM curve patterns can classify *P. insidiosum* into three assigned clades (i.e., A_TH_, B_TH_, and C_TH_). The limit of detection of qPCR-HRM is 1 pg of a genomic DNA template. 

Overall, qPCR has become a more popular amplification technique because it improves analytical sensitivity and reduces turnaround time. All processes of qPCR occur in a single, closed tube, thereby minimizing carry-over contamination and, thus, the acquirement of a false positive result. Apart from diagnostic applications, qPCR can be used in an epidemiological study of *P. insidiosum* (i.e., strain genotyping) and DNA quantity assessment (i.e., gene expression).

##### Isothermal Amplification

Although PCR-based amplification techniques have been widely used for various molecular analyses, they require dedicated thermocycler instrumentation with precise temperature control, limiting their usage in a resource-limited setting [8]. Over the past few decades, many non-PCR-based techniques have emerged as alternative nucleic acid amplification techniques performed under a single constant temperature to promote rapid and simple DNA amplification and detection, and with minimal tools required, for point-of-care uses [62]. Unlike the PCR assays, such an isothermal amplification method is carried out at a single working temperature, which can be set up in a water bath or heat block without requiring a thermocycler machine [63]. Compared with PCR, the isothermal amplification assay appears to be more tolerant against the reaction-inhibiting substances often present in biological samples, resulting in increased amplification efficiency [11]. The isothermal technology includes helicase-dependent amplification (HDA), loop-mediated isothermal amplification (LAMP), nucleic acid sequence-based amplification (NASBA), recombinase polymerase amplification (RPA), and strand displacement amplification (SDA) [11]. So far, HDA [45] and LAMP [39] have been applied for detecting *P. insidiosum*.

(1) Helicase-dependent amplification (HDA)

**Figure 8:**
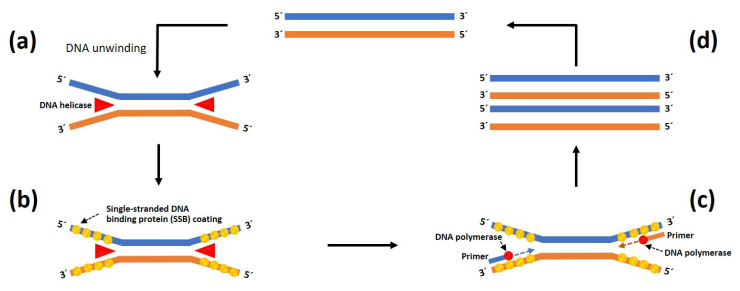
Schematic overview of the helicase-dependent amplification system used to detect *P. insidiosum*. (**a**) Target double-stranded DNA (dsDNA) is isothermally separated into single-stranded DNAs (ssDNA) by helicase and (**b**) stabilized by the single-stranded DNA-binding protein (SSB). (**c**) Two target sequence-specific primers anneal the 5′ and 3′ defined borders of the target ssDNA. Subsequently, DNA polymerase elongates the primer sequences to obtain new dsDNA amplification products. (**d**) This helicase-dependent amplification is repeated continuously, resulting in exponential amplification of the target DNA sequence. The diagram is adapted from the reference [66].

HDA is an in vitro isothermal nucleic acid amplification method first developed by Vincent et al. [64]. It has been adapted to detect various infectious agents [62]. This technique depends on the use of thermostable helicase activity to separate double-stranded DNAs without requiring a heat denaturation step. The single-stranded-DNA-binding proteins (SSB) are coated onto single-stranded DNA templates (ssDNA) to prevent the re-association of the complementary strand. Two designed primers anneal the 5′ and 3′ defined borders of the target ssDNA before proceeding with DNA polymerase-dependent extension to produce two copies of the target sequence. The process is repeated at a single temperature set in a heat block, resulting in the exponential amplification of the target DNA sequence (Figure 8) [64,65]. An HDA product can be detected on an electrophoresis gel as a size-specific band after it is stained with ethidium bromide or SYBR green. The detection of *P. insidiosum* using thermophilic HDA (tHDA) was first described by Worasilchai et al. [45]. Their assay employs two designed primers and an IsoAmp tHDA kit (BioHelix, Beverly, MA, USA) containing the thermostable UvrD helicase (for separating double-stranded DNAs) and thermostable DNA polymerase (for synthesizing DNA sequence) in order to multiply the target *cox*2 gene in a 2 hr amplification reaction at 65 °C. The resulting 91 bp amplicon was subjected to digestion with the restriction enzyme CviKI-1 to obtain 42 bp and 49 bp fragments—specific characteristics of *P. insidiosum*. tHDA has a detection limit of 100 fg (equivalent to 1 × 10^−4^ ng) of the genomic DNA template.

(2) Loop-mediated isothermal amplification (LAMP)

**Figure 9:**
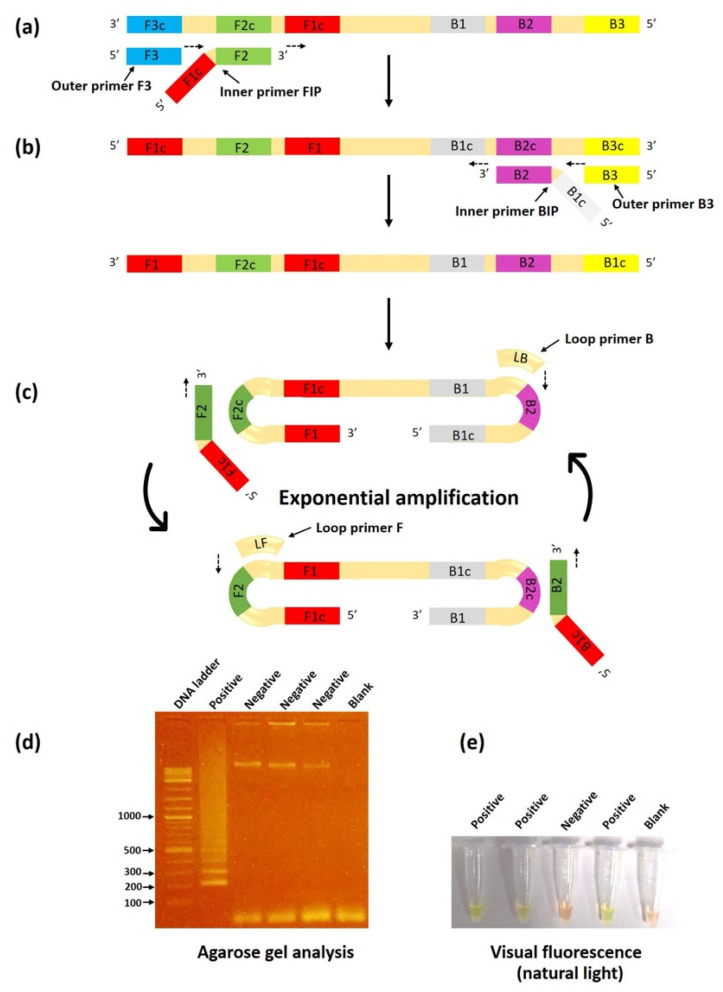
Schematic illustration of the LAMP assay for detecting *P. insidiosum*. (**a**) In the initial step of the LAMP method, the F2 portion of the FIP primer anneals the F2c region of the DNA template to form a new DNA strand using the *Bst* DNA polymerase. The F3 primer anneals the F3c region of the target DNA to initiate sequence elongation, causing displacement of the FIP-linked strand. (**b**) The displaced strand forms a loop at the 5’ end and subsequently serves as a template for DNA amplification using the primers BIP and B3. (**c**) As a result, a loop is formed at both ends. The obtained double stem-loop DNA (a dumbbell-like structure) enters multiple amplification cycles for exponential production of the target DNA. The final LAMP product is a mixture of various sizes of stem-loop DNAs. (**d**) The LAMP products can be detected as a ladder pattern observed on an electrophoresis agarose gel. (**e**) Naked-eye observation of an amplification result under natural light after adding a fluorescent intercalating dye (SYBR Green I) shows the striking color change from orange to bright green, indicating a positive LAMP reaction. The diagram is adapted from the references [68,69] and the New England Biolabs website (https://international.neb.com, accessed on 6 November 2022).

LAMP is a unique isothermal amplification technology developed by Notomi et al. [67]. This method is based on auto-cycling strand displacement DNA synthesis operated by the *Bst* DNA polymerase large fragment. The LAMP reaction occurs under isothermal conditions set between 60 and 65 °C for a specific duration (i.e., 60 min). Like the other isothermal amplification techniques, LAMP does not require a thermocycler for DNA denaturation, annealing, or extension, as is required for standard PCR-based assays [68]. The assay utilizes a set of four target-specific primers—comprising two outer primers (the forward outer primer F3 and the backward outer primer B3) and two inner primers (the forward inner primer FIP and the backward inner primer BIP)—to recognize six specific regions on the target sequence (Figure 9). The amplification speed can be accelerated by adding two loop primers: the forward loop primer LF and the backward loop primer LB. Cauliflower-like structures of multiple-loop DNAs and stem-loop DNAs with different inverted target repeats of the target sequence are the final LAMP product. A large quantity of the desired amplicons (i.e., 1000-fold higher than the conventional PCR) can be generated within 60 min by one complete LAMP reaction [68]. LAMP products can be detected in various ways, such as by the measurement of turbidity, colorimetric detection, agarose gel electrophoresis, real-time fluorescence, and a lateral flow assay [64].

LAMP is the most widely applied isothermal amplification technology for the rapid and straightforward identification of pathogenic microorganisms [69]. Htun et al. [39] employed the LAMP technique to detect the rDNA ITS sequence in *P. insidiosum*. Their LAMP products can be detected by observing SYBR Green illumination via the naked eye or by observing a ladder pattern of the amplicons on an electrophoresis gel. Compared with m-PCR [19], LAMP exhibited a 50% shorter turnaround time and a 10-fold lower detection limit (1 × 10^−4^ ng of genomic DNA template). Based on clinical sample evaluation, LAMP showed 88% sensitivity and 100% specificity. LAMP is another molecular assay that could be used to facilitate the early detection of *P. insidiosum* in resource-limited areas. 

##### Next-Generation Sequencing (NGS)

The emergence and application of NGS technology for obtaining microbial whole-genome sequences (WGSs) have markedly changed the approach to improving the clinical management of patients with infectious diseases [70]. As an advanced NAT, NGS has been used for microbial identification, employing an automated, throughput, and parallelized genome sequencer. The obtained sequences are compared and aligned with available genetic information from many organisms deposited in public databases, thereby aiding the identification of unknown microorganisms [71]. Moreover, WGS can provide detailed data for drug design, antimicrobial resistance, and gene mutation [72]. Some investigators have sequenced the genomes from various *P. insidiosum* strains to establish a genome database of this pathogen [73,74,75,76,77,78]. However, Zhang et al. are the first to adopt the NGS technique for identifying *P. insidiosum* in a severely ill patient with a suspected infectious condition, for which the standard microbial identification procedures failed to identify the etiological microorganism [79]. They employed the so-called metagenomic NGS (mNGS) and WGS to obtain microbial DNA data in clinical specimens (i.e., peripheral blood, skin tissue, and isolated microbial colony), searched the obtained sequences against the NCBI reference microbial database, and successfully identified the unknown pathogen as *P. insidiosum*. The mNGS is a novel, rapid, and robust method for microbial detection, especially in urgent clinical situations where the causative agent is unknown. However, this technology comes with a high cost and is not available in many laboratories, limiting its use, at the moment, as a routine microbial detection assay.

### 3.3. Advantages and Limitations of NATs for P. insidiosum Detection

Various NATs have been established for detecting *P. insidiosum* (Figure 2). Each technique has different diagnostic advantages and limitations, as summarized in Table 2 for a side-by-side comparison. For example, sequence homology analysis, a standard technique that uses universal fungal primers, can be performed in a general microbiological laboratory. However, this test is seriously time-consuming (i.e., days or weeks) as it requires a DNA-sequencing step. Unlike sequence homology analysis, conventional PCR (another standard molecular technique) requires *P. insidiosum*-specific primers (usually unavailable in general laboratories) that amplify a size-specific amplicon, bypassing the need for DNA sequencing. Nested PCR exhibits extremely high sensitivity, allowing it to detect a trace amount of the pathogen DNA in a clinical specimen. Recognized limitations of this assay include a longer turnaround time and a higher risk of carry-over contamination. Multiplex PCR can simultaneously amplify multiple target sequences, leading to the ability to detect and genotype a pathogen specifically. However, it tends to have a relatively lower detection sensitivity. Real-time PCR has high detection sensitivity and specificity, demonstrates the ability to check and quantify the target sequence in real time, and provides a short turnaround time. As a downside, like most PCR-based techniques, real-time PCR requires *P. insidiosum*-specific primers, and it is susceptible to an inhibitory substance in a test sample, not to mention the need for a more sophisticated and expensive machine. LAMP and had can detect *P. insidiosum* DNA without requiring a thermal machine to generate a test result. Nevertheless, their higher chance of carry-over contamination (in the case of LAMP) and non-specific products, expensive reagents, and susceptibility to an inhibitor in a test sample (in the case of HDA) limit the use of these isothermal assays. Recently, NGS has been reported as a novel and robust method for detecting *P. insidiosum*. It does not require a postulation for what is the most-likely pathogen, which is helpful for healthcare workers unfamiliar with *P. insidiosum*. The requirements for expensive equipment and reagents, skillful personnel, and extensive bioinformatic analysis prevent the clinical use of this technique.

## 4. Conclusions

Since the first report of NAT for the detection of *P. insidiosum* using sequence homology analysis in 2001, other NAT techniques have been implemented for the molecular diagnosis of pythiosis. However, the technical and clinical details on the use of NATs for detecting *P. insidiosum* are scattered, making it difficult for healthcare workers to learn and remain updated with respect to these techniques. Here, we performed a systematic review to collect and summarize the current information on the principles and applications of *P. insidiosum*-detecting NATs. Over the past two decades, many NAT techniques for the diagnosis of pythiosis have been reported in the literature and can be classified into non-amplification-based and amplification-based methods (Figure 2). The non-amplification-based NATs rely on DNA hybridization, a high-throughput method that can simultaneously detect multiple genes and samples. The amplification-based NATs can be subcategorized into PCR-based (i.e., sequence homology analysis, conventional PCR, nested PCR, multiplex PCR, and real-time PCR), isothermal amplification-based (i.e., HDA and LAMP), and NGS-based techniques. The assay principles of each NAT developed for *P. insidiosum* have been depicted in an easy-to-understand diagram (Figure 3, Figure 4, Figure 5, Figure 6, Figure 7, Figure 8 and Figure 9). As shown here, NATs can provide a timely and accurate diagnosis of pythiosis, leading to prompt treatment and a better prognosis for patients with pythiosis. NATs demonstrate several advantages compared with the other diagnostic modalities (i.e., microbial cultures and immunological assays), especially with respect to their ability to directly detect trace amounts of *P. insidiosum* in a sample with high detection sensitivity and specificity. However, the diagnostic advantages and limitations of each NAT technique compared with one another (Table 2) should be considered when selecting an appropriate test for clinical use in different laboratory environments. This review presents comprehensive but concise information on NATs for the molecular detection of *P. insidiosum*. It constitutes an up-to-date reference with which healthcare professionals can learn about the *P. insidiosum*-detecting NATs and thereby choose a suitable detection method according to their respective laboratory settings.

## Figures and Tables

**Table 1:** Primer sequences used in NATs for the detection of *P. insidiosum*.

Target Sequence	Primer ID	Primer Sequence (5′–3′ Direction)	References
ITS region	ITS1	TCCGTAGGTGAACCTGCGG	[16,17,18,19,20,21,22,23,24,25,26,27,28,29,30,31]
	ITS2	GCTGCGTTCTTCATCGATGC	[17,20,23,30]
	ITS2P	GCAGCGTTCTTCATCGATGT	[24]
	ITS2R ITS3	ATAACCAGCGTCCAGT TCG GCATCGATGAAGAACGCAGC	[22] [23,32,33]
	ITS4	TCCTCCGCTTATTGATATGC	[16,18,21,22,23,28,29,31,32,34]
	ITS5	GGAAGTAAAAGTCGTAACAAGG	[23,34]
	ITSpy1	CTGCGGAAGGATCATTACC	[29,35,36]
	ITSpy2	GTCCTCGGAGTATAGATCAG	[29,35,36]
	NE Fw	ATGCCTGGAAGTATGCCTGT	[32]
	NE Rev	TCACTGCGTTCGAGCATTAC	[32]
	TW81	GCGGATCCGTTTCCGTAGGTGAACCTGC	[37,38]
	AB28	GCGGATCCATATGCTTAAGTTCAGCGGGT	[37,38]
	PI1	TTCGTCGAAGCGGACTGCT	[16,17,22,24]
	PI2	GCCGTACAACCCGAGAGTCATA	[16,17,24]
	R1	CCTCACATTCTGCCATCTCG	[19,25,26,27]
	R2	ATACCGCCAATAGAGGTCAT	[19,25,26,27]
	R3	TTACCCGAAGGCGTCAAAGA	[19,25,26,27]
	ITS-F3	GGCAGAATGTGAGGTGTCTC	[39]
	ITS-B3	GGAAACAACACCCCGTCAG	[39]
	ITS-FIP	ACAGATCACTGCGTTCGAGCAT-TTTT-GGAGATAGCACGAGTCCCT	[39]
	ITS-BIP	TCAGATTGCTTTGCGCTGGTGG-TTTT-CCGAAGCCTAACATACCGC	[39]
18S rRNA	CPL6	GACACAGGGAGGTAGTGACAATAAATA	[7,40,41]
	CPR8	CTTGGTAAATGCTTTCGCCT	[7,40,41]
	NS1	GTAGTCATATGCTTGTCTC	[23,37,42,43]
	NS2	GGCTGCTGGCACCAGACTTGC	[23,37]
	NS8	TCCGCAGGTTCACCTACGGA	[23,42,43]
	Pin 1	TGGCTCTTCGAGTCGGGCAA	[35,36]
	Pin 2	GTCGGCATAGTTTATGGTTAAGA	[35,36]
	YTL1	CTTTGAGTGTGTTGCTAGGATG	[7,40,41]
	YTR1	CTGGAATATGAATACCCCCAAC	[7,40,41]
*cox*1	OomCoxI-Levup	TCAWCWMGATGGCTTTTTTCAAC	[20,44]
	OomCoxI-Levlo	CYTCHGGRTGWCCRAAAAACCAAA	[20,44]
*cox*2	Cox_Pi_5	TAATTTGGACTACTATTCCAGC	[45,46]
	Cox_Pi_6	GGATCAATGTATTTCATCCATAG	[45,46]
*exo*1	Dx3	GCGAGTTCTGGCTCGACTTTA	[47]
	Dx4	ACAAGCGCCAAAAAGTCCCA	[47]
	Pr77	AAGACGTACTACTGGAAG	[48]
	Pr78	CATAAAGTCGAGCCAGAA	[48]

**Table 2:** Advantages and limitations of each nucleic acid-base test for *P. insidiosum* detection.

Method	Advantages	Limitations	References
Dot-blot hybridization	- Simultaneously detects multiple genes at once - High throughput assay - Does not require DNA separation by electrophoresis	- Provides no information about the amplicon size - Time-consuming and multi-step procedure - Requires expensive reagents	[53,80,81,82]
Sequence homology analysis	- Does not require *P. insidiosum*-specific primers - Is feasible to perform in a general molecular laboratory	- Requires universal fungal primers - Time-consuming and multi-step procedure - Requires expensive equipment and reagents - Requires a highly purified PCR product for sequencing - Requires a DNA-sequencing step	[5,83]
Conventional PCR	- Primer’s design is simple - Simple procedure	- Requires expensive equipment and reagents - Requires post-amplification gel electrophoresis - Susceptible to inhibitory substances present in a given test sample	[11,84,85]
Nested-PCR	- High detection sensitivity and specificity - Reduces appearance of non-specific products by second-round amplification - Ability to detect a microorganism present in a low quantity	- Requires expensive equipment and reagents - Time-consuming (requiring 2 sequential amplification reactions) - High risk of carry-over contamination - Susceptible to an inhibitory substance present in a test sample	[7,14,57]
Multiplex PCR	- Simultaneously detects multiple genes/sequences at once - Ability to genotype *P. insidiosum*	- Primer design is complex - Requires PCR condition optimizations - Relatively low detection sensitivity - Susceptible to an inhibitory substance present in a test sample	[8,11,61]
Real-time PCR	- High detection sensitivity and specificity - Provides a quantitative result - Provides a real-time result during the PCR process - Low chance of carry-over contamination - High throughput assay and short turnaround time - Does not require post-amplification gel electrophoresis	- Requires expensive equipment and reagents - Provides no information about the amplicon size - Detects non-specific PCR products when using a non-specific dye - Susceptible to an inhibitory substance present in a test sample	[8,11,14,15]
Helicase-dependent amplification (HDA)	- Does not require a thermocycler - Primer design is simple	- Yields non-specific products from mispriming and primer dimers - Requires expensive reagents - Susceptible to an inhibitory substance present in a test sample	[63,65]
Loop-mediated isothermal amplification (LAMP)	- Does not require a thermocycler - High detection specificity - Simple and low-cost procedure - Short assay duration (~30 min) - Direct result readout observable by the naked eye - Tolerant to an inhibitory substance present in a test sample	- Primer design is complex - Prone to carry-over contamination - No information about the amplicon size	[62,69,86]
Next-Generation Sequencing (NGS)	- Novel, rapid, and robust method for microbial detection - Does not require formation of hypothesis regarding the most-likely pathogen	- Requires expensive equipment and reagents - Assay is unavailable in many laboratories - Requires experienced personnel and exhaustive bioinformatic analysis	[79]

## Data Availability

Not applicable.
